# Prevalence of anaemia and low intake of dietary nutrients in pregnant women living in rural and urban areas in the Ashanti region of Ghana

**DOI:** 10.1371/journal.pone.0226026

**Published:** 2020-01-24

**Authors:** Jessica Ayensu, Reginald Annan, Herman Lutterodt, Anthony Edusei, Loh Su Peng

**Affiliations:** 1 Department of Biochemistry and Biotechnology, Kwame Nkrumah University of Science and Technology, Kumasi, Ghana; 2 Department of Clinical Nutrition and Dietetics, University of Cape Coast, Cape Coast, Ghana; 3 Department of Food Science and Technology, Kwame Nkrumah University of Science and Technology, Kumasi, Ghana; 4 School of Public Health, Kwame Nkrumah University of Science and Technology, Kumasi, Ghana; 5 Department of Nutrition and Dietetics, Universiti Putra, Selangor, Malaysia; CSIR-Foood Research Institute, GHANA

## Abstract

**Background:**

Anaemia remains a major cause of morbidity and mortality among women and children worldwide. Because deficiencies in essential micronutrients such as iron, folate and vitamin B12 prior to and during gestation increase a woman’s risk of being anaemic, adequate dietary intake of such nutrients is vital during this important phase in life. However, information on the dietary micronutrient intakes of pregnant women in Ghana, particularly of those resident in rural areas is scanty. Thus, this study aimed to assess anaemia prevalence and dietary micronutrient intakes in pregnant women in urban and rural areas in Ghana.

**Methods:**

A comparative cross sectional study design involving 379 pregnant women was used to assess the prevalence of anaemia and low intake of dietary nutrients in pregnant women living in rural and urban areas in the Ashanti region of Ghana. Anaemia status and mid upper arm circumference (MUAC) were used as proxy for maternal nutritional status. Haemoglobin measurements were used to determine anaemia prevalence and the dietary diversity of the women were determined with a 24-hour dietary recall and a food frequency questionnaire.

**Results:**

Overall, anaemia was present in 56.5% of the study population. Anaemia prevalence was higher among rural residents than urban dwellers. Majority of the respondents had inadequate intakes of iron, zinc, folate, calcium and vitamin A. The mean dietary diversity score (DDS) of the study population from the first 24-hour recall was 3.81 ± 0.7. Of the 379 women, 28.8% met the minimum dietary diversity for women (MDD-W). The independent predictors of haemoglobin concentration were, gestational age, maternal age and dietary diversity score. Such that respondents with low DDS were more likely to be anaemic than those with high DDS (OR = 1.795, p = 0.022, 95% CI: 1.086 to 2.967).

**Conclusions:**

A large percentage of pregnant women still have insufficient dietary intakes of essential nutrients required to support the nutritional demands during pregnancy. Particularly, pregnant women resident in rural areas require interventions such as nutrition education on the selection and preparation of diversified meals to mitigate the effects of undernutrition.

## Introduction

Regardless of the many life-threatening challenges a woman experiences, the news of pregnancy always brings joy to her and her family. This period of dynamic change requires an expectant mother to pay critical attention to her health to ensure desirable birth outcomes[1, 2]. Because a developing foetus relies solely on its mother for nourishment, adequate maternal nutrition is vital for proper maternal health. Poor maternal nutritional status has been associated with a myriad of adverse maternal and foetal outcomes like maternal and perinatal mortality, low birth weight, pregnancy induced hypertension, intrauterine growth restrictions and gestational diabetes and foetal programming [3,4].

According to a joint report from the United Nations Population Fund (UNFPA), World Health Organisation (WHO), United Nations Children’s Education Fund (UNICEF) and the World Bank, over 800 women die daily from pregnancy complications and childbirth with 99% of these mortalities occurring in developing countries. Anaemia is an eminent cause of morbidity and mortality among pregnant women and about 40% of all maternal deaths during parturition is attributed to it [5, 6]. Defined as low blood haemoglobin concentration, anaemia continues to be a major issue of public health concern in many developing countries [7]. Although it affects people at different stages of the life cycle, anaemia is most prevalent among pregnant women and children under 5 years old. Anaemia is generally associated with negative health consequences and affects social and economic development[8].

Among the many identified nutritional causes of anaemia, iron deficiency is the most common contributor accounting for approximately 50% of all global cases and anaemia incidence in pregnancy. Iron deficiency usually results from low dietary iron intake and poor iron bioavailability because of over-reliance on plant-based diets high in inhibitors of iron absorption such as phytate [9]. During pregnancy, the increased physiological changes exert a demand for additional iron. Infections such as malaria, hook-worm and helminths are well-known in the pathogenesis of anaemia in pregnancy[10]. Consequences of anaemia include reduced work capacity and increased risk of mortality for the mother, and premature delivery, low birth weight and poor mental development for the infant[11].

Additionally, maternal anthropometry, specifically underweight and overweight are important risk factors for undesirable pregnancy and birth outcomes. According to [3], pregnant women who are obese have an increased risk of fetal macrosomia, low Apgar scores and perinatal death and have a four times probability of developing gestational diabetes and two times chance of developing hypertensive disorders compared with women with normal weight. Maternal underweight increases the risk of intra uterine growth restrictions. Over the past 10 years, trends in weight of women age 15 to 49 has changed towards overweight and obesity [12]. In 2014, the Ghana statistical service reported that 25% of women are overweight and 15% are obese[13].

In Ghana, interventions such as free provision of mosquito nets, health and nutrition education during antenatal clinics, screening and treatment of anaemia, hypertensive disorders and diabetes, providing multiple vitamins and mineral supplements, weight monitoring and intermittent prophylaxis treatment for malaria (IPTp) with sulfadoxine pyrimethamine from the second trimester have been intensified to reduce the burden of malnutrition in pregnant women [13]. In spite of all these interventions, maternal malnutrition is still a force to be reckoned with in Ghana. Identifying and providing sustainable solutions to the causes of maternal malnutrition is essential for ensuring desirable outcomes of pregnancy. However, majority of studies aimed at identifying causes of malnutrition in pregnancy have focused on specific categories of factors. Moreover, there is paucity of scientific data on the nutritional status of pregnant women resident in rural communities in the country although they are highly susceptible to malnutrition due to food insecurity and inability to access antenatal care and supplementation and fortification programs as a result of poor access routes. This present study aimed to assess the prevalence and severity of undernutrition among pregnant women in rural and urban areas in four districts in the Ashanti region of Ghana.

## Subjects and methods

### Ethics

The protocol for the study was reviewed and approved by the Committee on Human Research, Publications and Ethics, of the School of Medical Sciences, Kwame Nkrumah University of Science and Technology (CHRPE/KNUST) Kumasi, Ghana. A written permission to undertake the study was obtained from district health directors and the medical directors of the health centres. Participation in the study was voluntary and pregnant women who agreed to participate in the study were made to thumb print or append their signatures on a participant consent form in the presence of a witness.

### Study area

The study was undertaken in seven communities in four districts in the Ashanti Region of Ghana. Of the 16 administrative regions in Ghana, the Ashanti region is the most populated with a population of 4,780,380 and almost half of the inhabitants residing in rural areas. According to the 2014 Demographic Health Survey conducted by the Ghana Health Services (GHS), about 111,059 children age 6 to 59 months suffer from stunted growth, the second highest after the Northern Region, which recorded 118,427 cases [13]. According to the same report, the study area has the second highest (45.4%) obesity prevalence among reproductive age women in the country. Regarding anaemia, the prevalence (40.5) is higher than the prevalence in Upper West region (35.6%) and Upper East region (39.6%), which are known for high poverty prevalence. These indicate that the study area is plagued with malnutrition. The majority of people are engaged in agriculture whilst some are involved in trading. The main staple foods include cassava, plantain, rice, yam, cocoyam and maize. The region experiences double maxima rainfall in a year, with peaks in May/June and October. Mean annual rainfall is between 1100 mm and 1800mm.

### Study design, population and sampling

The study was a quantitative cross-sectional survey comprising 379 pregnant women attending ante natal clinics in seven health centres in rural and urban areas in the Ashanti region of Ghana from March 2017 to June 2017. The simple random sampling technique was used to recruit respondents. Pregnant women recruited into the study were aged 15 to 49 years and were attending antenatal clinic for the first time at the selected health centres over the study period. All women who had a singleton pregnancy without medical complications were identified with the help of study nurses during registration and invited to participate in the study. Pregnant women were exempted from the study if they had a multiple birth or complicated pregnancy.

The sample size was determined based on anaemia prevalence among women in the Ashanti region 40.5% [13] with a marginal error of 5%, 95% confidence interval (CI) and an attrition rate of 5%. Based on the identified prevalence, the sample size for the study was calculated using the Cochrane formulae for cross-sectional studies [14].

$$N=\frac{\;Z^{2}p(1-p)}{d^{2}}$$

Where N is the sample size, Z is the z-score (1.96), p is the estimated prevalence in the study area and d the marginal error at 5% (0.05). This resulted in a sample size of 388. Seven health care centres were randomly selected from the four districts. Based on the calculated sample size, 388, a quota of 55 was assigned to each health centre. Afterwards, systematic random sampling was used to select study participants. After the first pregnant woman was randomly selected, a sampling interval of 3 was used to recruit 55 pregnant women from each health centre.

### Data collection

A pretested structured questionnaire with closed and open-ended questions was used to obtain information on socio-demographic characteristic; obstetric data; medical history and nutritional status (dietary intakes, anthropometric status and biochemical assessment). The primary outcomes of concern were anaemia and maternal mid-upper arm circumference (MUAC). The questionnaire for this study was pretested and validated to make sure the questions elicited the expected responses. The covariate variables included maternal age, gestational age at recruitment, maternal height, dietary intakes, dietary diversity score, marital status, residence, occupation and highest educational level attained.

### Anthropometric assessment

At recruitment, anthropometric measurements which included height, weight and MUAC were taken by trained research assistants. The height was measured to the nearest 0.1 cm without shoes with a Seca stadiometer (CE0123). Weight measurements were done with a body composition analyser (Omron Karada Scan, BF508) and recorded to the nearest 0.1kg. The mid upper arm circumference measurement was done by locating the midpoint between the acromion and olecranon process on the left hand of participants. This was measured to the nearest 0.1cm. All anthropometric measurements were done in duplicates. Pregnant women with MUAC values below 25.0cm were categorized as having low body weight [15]. Although the weight of the participants was taken at recruitment, the MUAC was used as proxy for maternal body weight since it is rarely affected by gestational age.

### Biochemical assessment

At recruitment, 2mL of venous blood were collected from each participant into EDTA anticoagulant tubes and used for haemoglobin assay. The blood samples were transported from study sites to the laboratory in ice chests containing ice packs. The haemoglobin assay was done using Sysmex Haematology System (USA) at the Clinical Analysis Laboratory of the Department of Biochemistry and Biotechnology, Kwame Nkrumah University of Science and Technology with. The WHO cut off for determining anaemia in pregnant women, haemoglobin levels less than 11 g/dL, was used for determining anaemia in this study and anaemia was further classified as mild (9.0–10.9 g/dL), moderate (7.0–8.9 g/dL), or severe (<7.0 g/dL). Additionally, the WHO classifies anaemia as severe when the prevalence was 40% or more in any group (all types of anaemia) or when severe anaemia (haemoglobin < 7 g/dL) exceeds 2%.

### Dietary assessment

Three 24-hour recall sessions were conducted to obtain information on the daily nutrient intakes of pregnant women included in the study. In a face to face interview, study participants were asked to recall food and beverage intake of previous 24hours particularly, two weekdays and one weekend. After the recall, participants were asked to estimate their actual intakes with the aid of common household handy measures and food models. The estimated intakes were converted to grams with an already prepared food conversion. Daily dietary nutrient intakes of the pregnant women were estimated using the Nutrient Analysis Template (Department of Food Science and Nutrition, University of Ghana) and the 2012 West African Food Composition Table. The nutrient analysis template was also used to generate the dietary diversity score of the study subjects. The recall days were Monday, Friday and Sundays. The 24-Hour recall was used to classify participants as having adequate or inadequate intakes according to RDA for pregnant women.

### Assessment of dietary diversity

The general dietary quality of the respondents was assessed using the individual dietary diversity since it is a reliable indicator of nutrient adequacy and can be efficiently used as a measure of nutrient adequacy among pregnant females. The Women’s dietary diversity scores (WDDSs) were calculated based on a 24-hour dietary recall period and the number of food groups consumed within the period. The food groups used were all starchy staples, dark green leafy vegetables, eggs, legumes, nuts and seeds, milk and milk products, meat and fish, organ meats, fruits and vegetables and other vitamin A rich fruits and vegetables and red palm oil. Women who obtained a diversity score of less than 5 were classified as having low dietary diversity and scores of 5–10 were classified in the high dietary diversity.

### Statistical analysis

Data was analyzed using IBM SPSS Statistics 23 software. The data was filtered after entry to ensure there were no missing values and outliers. Independent sample T-test was used to compare group mean differences. Association between MUAC, anaemia and some predictor variables was tested using chi-square and Fisher’s Exact Test. Variables with p values less than 0.05 were entered into binary logistics regression model. Independent variables included in the regression model regress were marital status, residence, dietary vitamin B3 and B6 intakes, dietary diversity score and gestational age. And the independent variables were anaemia status. Categorical variables were coded and used for the analysis. Differences and associations were considered statistically significant at *p* < 0.05.

## Results

### Socio-demographic characteristics of the sample

The socio-demographic characteristics of the respondents are presented in Table 1. A total of 379 pregnant mothers agreed to participate in the study. More than half (59.1%) of the participants were resident in urban areas whiles the remaining 40.9% were resident in rural areas. The mean age was 28.34±0.32 years, ranging from 15 to 45years. Majority (85.8%) of the respondents were Christians. With regards to gestational age, majority (43.6%) of the mothers were in their second trimester. Petty trading was common among the respondents (30.6%) and 17.4% were engaged in other occupations such as teaching, nursing, catering and civil service. More than half (66.2%) of the respondents were married. The highest educational level attained by most (47%) of the women was junior high with tertiary education being the least (8.2%) attained.

**Table 1:** Socio-demographic characteristics of the sample.

Variable	Frequency n (%)	Rural (%)	Urban (%)
**Age groupings (years)**			
15–24	109 (28.8)	57.8	42.2
25–34	205 (54.1)	37.1	62.9
35–49	65 (17.1)	16	49
**Religion**			
Christian	325 (85.8)	40.9	59.1
Muslim	50 (13.2)	42	58
Other	4 (1)	0	100
**Classification of Occupation**			
Farmer	41 (10.8)	97.6	2.4
Trader	116 (30.6)	33.6	66.4
Hair dresser	46 (12.1)	34.8	65.2
Tailor	34 (9)	38.2	61.8
Student	4 (1.1)	75	25
Other	66 (17.4)	25.8	74.2
Unemployed	72 (19)	37.5	62.5
**Educational Level**			
None	44 (11.6)	50	50
Primary	44 (11.6)	52.3	47.7
Junior High School	178 (47)	48.9	51.1
Senior High School	82 (21.6)	23.2	76.8
Tertiary	31(8.2)	12.9	87.1
**Marital status**			
Single	76 (20.1)	85.5	14.5
Married	251 (66.2)	32.3	67.7
Widowed	1 (0.3)	100	0
Cohabiting	48 (12.6)	14.6	85.4
Separated	3 (0.8)	33.3	66.7
**Gestational Age**			
First Trimester	77 (20.3)	72.7	27.3
Second Trimester	165 (43.6)	48.5	51.5
Third Trimester	137 (36.1)	13.9	86.1

Categorical data are presented as percentages. Other occupation represents teaching, nursing, catering and civil service.

### Dietary intakes of respondents

Table 2 provides a general overview of the mean daily dietary intake of the study participants. Overall, the total mean intakes for selenium (65.63±1.55mcg), vitamin C (103.52±3.10 mg), vitamin B12 (3.69±0.49mcg) and carbohydrate (267.42±6.29g) were higher than the RDA for the nutrients. Additionally, an independent sample T-test performed showed no significant difference between the dietary intake for iron, vitamin A, vitamin B6, vitamin B12, vitamin B2, folate, vitamin C, and vitamin E among urban and rural dwellers. The mean protein intakes of participants resident in urban (53.7±1.49g) areas was significantly higher than that for rural residents (43.93±1.38g). The mean zinc intake for rural residents (6.03±0.2g) was significantly lower than that for the urban residents (8.06±0.26mg). The mean dietary diversity score (DDS) of the study population was 3.81±0.7. Out of 379 women, 28.8% met the Women Minimum Dietary Diversity Score (MDD-W).

**Table 2:** Mean daily nutrient intakes of respondents from repeated 24 Hour Recalls.

NUTRIENT	RDA	Overall mean (±SEM)	Rural (mean±SEM	Urban (mean±SEM)
**Carbohydrate**	175g/d	267.42±6.29	234.54±7.43^a^	290.17±9.02^b^
**Protein**	71g/d	49.62±1.07	43.93±1.38^a^	53.78±1.49^b^
**Iron**	27mg/d	10.94±0.63	10.84±1.45^a^	11.01±0.33^a^
**Vitamin A**	770mcg/d	181.34±7.27	171.40±8.91^a^	188.22±10.64^a^
**Vitamin B1**	1.4mg	1.01±0.03	0.93±0.03^a^	1.07±0.04^b^
**Vitamin B2**	1.4mg/d	0.95±0.03	0.84±0.11^a^	1.02±0.04^a^
**Vitamin B3**	18mg	14.4±0.44	12.45±0.47^a^	15.84±0.65^b^
**Vitamin B6**	1.9mg/day	2.03±0.45	2.55±1.10^a^	1.67±0.06^a^
**Vitamin B12**	2.6mcg/d	3.69±0.49	4.54±1.16^a^	3.11±0.19^a^
**Folate**	600mcg	296.02±8.68	280.10±11.22^a^	307.04±12.43^a^
**Vitamin C**	85mg/d	103.52±3.10	105.61±4.81^a^	102.08±4.81^a^
**Vitamin E**	15mg/d	6.86±0.21	7.31±0.34^a^	6.55±0.27^a^
**Zinc**	12mg/d	7.23±0.18	6.03±0.20^a^	8.06±0.26^b^
**Calcium**	1300mg/d	271.31±7.39	232.37±9.09^a^	298.25±10.45^b^
**Selenium**	60mcg/d	65.63±1.55	58.24±1.94^a^	70.78±2.19^b^

Data presented are the results of three 24hour recalls. Continuous data are presented as means. Continuous data were compared using paired t-test: means with the same superscript (alphabets) are not statistically different. RDA—Recommended Dietary Allowance, mcg/d- Microgram per Day, mg/d—Milligram per Day P<0.05.

### Prevalence of nutrient inadequacies among respondents

The distribution of respondents according to their nutrient intakes in relation to the RDAs are presented in Table 3. Majority of the study population had inadequate intakes of protein (85.2%), iron (99.2%), vitamin A (99.7%), vitamin B1 (83.9%), vitamin B2 (86.5%), folate (71.5%), vitamin B3 (80%), vitamin B6 (74.1%), zinc (90.2%), calcium (90.2%) and vitamin E (94.7%). Maternal nutrient inadequacies for all nutrients except vitamin C and calcium were higher among rural residents than urban dwellers.

**Table 3:** Percentage of pregnant women with nutrient adequacies and anadequacies.

Nutrient	RDA	Total n (%)	Urban n (%)	Rural n (%)
**Carbohydrate**	175g/d			
Adequate		299 (78.9)	184 (82.1)	115 (74.7)
Inadequate		80 (21.1)	40 (17.9)	40 (25.3)
**Protein**	71g/d			
Adequate		56 (14.8)	44 (19.6)	12 (7.7)
Inadequate		323(85.2)	180 (80.4)	143 (92.3)
**Iron**	27mg/d			
Adequate		3 (0.8)	2 (0.9)	1 (0.6)
Inadequate		376 (99.2)	222 (99.1)	154 (99.4)
**Vitamin A**	770mcg/d			
Adequate		1 (0.3)	1 (0.4)	0 (0)
Inadequate		378 (99.7)	223 (99.6)	155 (100)
**Vitamin B1**	1.4mg			
Adequate		61 (16.1)	44 (19.2)	17 (10.5)
Inadequate		318 (83.9)	181 (80.8)	137 (89.5)
**Vitamin B2**	1.4mg/d			
Adequate		51 (13.5)	41 (18.3))	10 (6.5))
Inadequate		328 (86.5)	183 (81.7)	145 (93.5)
**Vitamin B3**	18mg			
Adequate		91 (24)	65 (29)	26 (17)
Inadequate		288(80)	159 (71)	129 (83)
**Vitamin B6**	1.9mg/day			
Adequate		98 (25.9)	70 (31.3)	28 (18.1)
Inadequate		281 (74.10	154 (68.7)	127 (81.9)
**Vitamin B12**	2.6mcg/d			
Adequate		206 (54.4)	124 (55.4)	82 (52.9)
Inadequate		173 (45.6)	100 (44.6)	73 (47.1)
**Folate**	600mcg			
Adequate		21 (5.5)	16 (7.1)	5 (3.2)
Inadequate		271 (71.5)	208 (92.9)	150 (96.8)
**Vitamin C**	85mg/d			
Adequate		208 (54.9)	116 (51.8)	92 (59.4)
Inadequate		171 (45.1)	108 (48.2)	63 (40.6)
**Vitamin E**	15mg/d			
Adequate		20 (5.3)	11 (4.9)	9 (5.8)
Inadequate		359 (94.7)	213 (95.1)	146 (94.2)
**Zinc**	12mg/d			
Adequate		37 (9.8)	32 (14.3)	5 (3.2)
Inadequate		342 (90.2)	192 (85.7)	150 (96.8)
**Calcium**	1300mg/d			
Adequate		0 (0)	0	0
Inadequate		379 (100)	224 (100)	155 (100)
**Selenium**	60mcg/d			
Adequate		204 (53.8)	136 (61)	68 (44.2)
Inadequate		175 (46.2)	88 (39)	87 (55.8)

Categorical data are presented as percentages. RDA—Recommended Dietary Allowance, mcg/d- Microgram per Day, mg/d—Milligram per Day.

### Nutritional status of pregnant women

Based on the MUAC cutoffs, the overall prevalence of underweight (MUAC <25.0cm) was 10.6%, whiles 89.4% were classified as normal (MUAC ≥25.0cm). The mean MUAC was 29.05±0.01cm. Maternal underweight was more prevalent among pregnant women resident in rural areas (14.2%) than those in urban areas (8%). The mean haemoglobin concentration among the pregnant women studied was 11.01±0.31g/dl. The overall prevalence of anaemia was 56.5%. In terms of severity, mild anaemia was 87.9%, moderate anaemia was 9.8% and severe anaemia was 2.3%. Additionally, anaemia was more prevalent in rural areas (67.1%) than in urban areas (49.1%). All but 0.4% of the respondents had normal height (Table 4).

**Table 4:** Prevalence of anaemia and nutritional status of sregnant women in the Ashanti Region, Ghana.

Variable	Overall Total	Urban% (n)	Rural % (n)	p-value
**Haemoglobin Concentration (g/dl)**				
Mean	11.01±0.31	11.4±0.51^a^	10.44±0.10^a^	0.067
Normal	43.50%	50.9 (114)	32.9 (51)	
Anaemia	56.50%	49.1 (110)	67.1 (104)	
**Anaemia Classification**				
Mild Anaemia	87.90%	90 (99)	85.6 (89)	
Moderate Anaemia	9.80%	8.2 (9)	11.5 (12)	
Severe Anaemia	2.30%	1.8 (2.6)	2.9 (3)	
**MUAC (cm)**				
Mean	29.05±0.01	29.65±0.29^a^	28.19±0.29^b^	0.001
Normal	89.40%	92 (206)	85.8 (133)	
Underweight	10.60%	8 (18)	14.2 (22)	
**Dietary Diversity Score**				
Mean	3.81±0.7	4.33±0.81^a^	3.05±0.01^b^	0
Low	71.20%	55.32 (135)	87.09 (135)	
High	28.80%	39.7 (89)	12.90 (20)	

Categorical data are presented as percentages. Means with the same superscript (alphabets) are not statistically different.

### Factors associated with maternal nutritional status

The relationship between anaemia and maternal socio-demographic characteristics, dietary intake and dietary diversity was investigated. Chi-square analysis revealed positive association between anaemia among respondents and gestational age, DDS (dietary diversity score), maternal age, vitamin B3 intake, vitamin B6 intake and residence (Table 5). The significant determiners of maternal MUAC from the analysis were marital status, maternal age and iron intake (Table 6).

**Table 5:** Bivariate analyses of factors associated with anaemia of pregnant women (*n* = 379).

Characteristic	N	Anaemia		Test Statistics
		No	Yes	
		n (%)	n (%)	
**DDS**				
Low	270	101 (37.4)	169 (62.6)	χ^2^ = 14.3, p<0.0001
High	109	64 (58.7)	45 (41.3)	
**Gestational Age**				
First trimester	77	32 (41.6)	45 (58.4)	
Second trimester	165	53 (32.1)	112 (67.9)	χ^2^ = 21.2, p<0.0001
Third trimester	137	80 (58.4)	57 (41.6)	
**Maternal Age (years)**				
15–24	109	32 (29.4)	77 (70.6)	χ^2^ = 13.4, p = 0.004
25–34	204	101 (49.5)	103 (50.5)	
35–49	65	31 (47.7)	34 (52.3)	
**Vitamin B3 Intake**				
Adequate	91	48 (52.7)	43 (47.3)	χ^2^ = 4.3, p = 0.038
Inadequate	288	115 (39.9	173 (60.1)	
**Vitamin B6 Intake**				
Adequate	98	51 (52)	47 (48)	χ^2^ = 3.9, p = 0.047
Inadequate	281	113 (40.2)	168 (59.8)	
**Residence**				
Rural	155	51 (32.9)	104 (67.1)	χ^2^ = 12.1, p = 0.001
Urban	224	114 (50.9)	110 (49.1)	

Categorical data presented as percentages, Chi-Square test of association. P< 0.05. DDS = Dietary Diversity Score.

**Table 6:** Bivariate analyses of factors associated with MUAC of pregnant women (*n* = 379).

Characteristic	N	MUAC		Test Statistics
		Low	High	
		n (%)	n (%)	
**Marital Status**				
Single	76	13 (17.1)	63 (82.9)	
Married	251	18 (7.2)	233 (92.8)	χ2 = 17.2, p = 0.002
Widowed	1	1 (100)	0 (0)	
Cohabiting	48	8 (16.7)	40 (83.3)	
Separated	3	0 (0)	3 (100)	
**Age (years)**				
15–24	109	18 (16.5)	91 (83.5)	χ2 = 12.0, p = 0.008
25–34	204	22 (10.8)	182 (89.2)	
35–49	65	1 (1.5)	64 (98.5)	
**Iron Intake**				
Adequate	3	2 (66.7)	1 (33.3)	χ2 = 10.1, p = 0.030*
Inadequate	376	38 (10.1	338 (89.9)	

Categorical data presented as percentages, Chi-Square test of association.

*Fisher’s exact test. P< 0.05. DDS = Dietary Diversity Score.

A binary logistics regression was performed to ascertain the determinants of anaemia status of the respondents based on variables identified from correlation analysis. Possible explanatory variables that were tested but found insignificant were marital status, residence, and vitamin B3 and B6 intake. The significant independent predictor variables of anaemia status were maternal dietary diversity score, maternal age and gestational age.

In view of the odds ratio, respondents with low DDS were more likely to be anaemic than those with high DDS (OR = 1.795, p = 0.022, 95% CI: 1.086 to 2.967). Pregnant mothers who were in their second trimester of pregnancy had a significant chance of being anaemic than those in their first and third trimesters (OR = 2.065, p = 0.006, 95% CL: 1.228 to 3.470). For maternal age, respondents within the age bracket of 15 to 24 years were more likely to be anaemic than those within the bracket 25 to 34 years and 35 to 49 years (OR = 2.397, p = 0.028, 95% CI: 1.097 to 5.240). As regards maternal MUAC, none of the predictor variables identified from the chi-square analysis was found to have significant effects (Table 7).

**Table 7:** Determinants of anaemia in pregnant women (binary logistics regression analysis).

Model	OR (Odds Ratio)	Sig.	Confidence Interval	
			Lower Bound	Upper Bound
**DDS**	1.795	0.022	1.086	2.967
**Gestational Age**				
**First Trimester**	0.895	0.753	0.45	1.781
**Second Trimester**	2.065	0.006	1.228	3.47
**Maternal Age**				
**15–24 years**	2.397	0.028	1.097	5.24
**25–34 years**	1.112	0.738	0.598	2.068

DDS = Dietary Diversity Score, P<0.05

## Discussion

The nutritional status of a woman prior to, during and after pregnancy is critical for desirable outcomes. The determinants of maternal nutritional status are multifactorial and the contribution of each of the factors varies by dietary practice, geographical location, socio-demography and season. The aim of the study was to determine the factors that are associated with the nutritional status of pregnant women in rural and urban areas in the Ashanti region of Ghana. The findings of the study suggest that maternal age, dietary diversity score and gestational age are significant determiners of anaemia among pregnant women in the study. Potential explanatory variables for MUAC size were found to be insignificant.

### Dietary intakes of pregnant women

Data obtained from the 3-day 24hour recall questionnaire was used to estimate the absolute dietary nutrient intakes of respondents and compared to the recommended allowances for pregnant women. The findings of the study showed that, over 50% of the pregnant women were at risk of having inadequate daily intakes of vitamins A, E, B2, B3, B6, folate, iron, protein, calcium and zinc. In addition, no significant difference was observed between the dietary intakes of iron, folate, vitamins B12, B2, B6, C, E and folate of participants resident in rural areas and those in urban areas.

The average dietary diversity score for the women in the study (3.81 ± 0.7) was lower than the minimum dietary diversity score recommended for pregnant women. The low DDS and the inadequate dietary intakes for most of the nutrients among the pregnant women is an indication that the women are unlikely to meet their nutrient requirements needed to support the growth and the development of their foetus. Pregnant women need to eat a wide variety of foods to improve their nutrition and also prevent adverse outcomes associated with malnutrition in pregnancy.

The results of this study are similar to findings of [16] who reported low DDS (4.2±1.5) among pregnant women in rural areas in the three Northern regions in Ghana and that of the Ghana Micronutrient survey 2107 which reported an average DDS of 4.4 among pregnant women in the country [15]. Boke [17] also reported that lactating mothers resident in rural areas were 3.1 times more likely to have low dietary diversity as compared to those living in urban areas [17]. Comparison of dietary diversity scores between residences of respondents in this study revealed that the DDS for rural residents was significantly lower than the DDS for urban dwellers. People often believe that because of their involvement in agricultural activities, individuals resident in rural areas have access to a wide variety of fresh and nutritious foods and thus will have high dietary diversity. However, this is notion has often been proven wrong. Individuals resident in urban areas have been reported to have higher DDS than those in rural areas. This could be due to the fact that appropriate nutritional knowledge is needed for the selection and preparation of diverse foods, however, majority of rural dwellers have low educational attainment [13] and may lack knowledge on how to combine different foods to achieve optimum nutrition. Again, food and nutrition education is a component of the Basic school curriculum in Ghana and those who complete such levels are most likely to have higher nutritional knowledge than those who do not. For instance, Boke [17] found that lactating mothers who were illiterate were 2.5 times more likely to have low dietary diversity.

Additionally, not all rural residents engage in agricultural practices and may not have access to a variety of foods. Ecker [18] reported that farm households in rural areas in Ghana have a slightly higher dietary diversity than those who are not because of direct access to food due to farming [18]. Likewise, Bhagowalia [19] also reported that cattle or buffalo ownership improved milk consumption among children in rural Indian households [19]. It can thus be hypothesized that residence in a rural farming community is not an indication of a high DDS. Furthermore, high levels of poverty in rural areas may affect the food purchasing power of rural residents [20].

### Prevalence of undernutrition

Overall, anaemia was found to be present in 56% of the pregnant women studied and can be classified as a severe public health problem according to WHO guidelines [5]. This is within the 51.9–59.6% estimated prevalence of anaemia in pregnancy in Africa [21]. But higher than what is reported in the 2014 Ghana Demographic and Health Survey and Ghana Micronutrient survey in which, overall, 44.6% and 42% of pregnant women in Ghana suffered from some degree of anaemia respectively[13, 15]. The mean MUAC of the respondents was 29.05±0.01cm. This was higher than the 28.6cm reported from the Ghana Micronutrient Survey 2017. In this study, only 10.6% of the respondents had a MUAC that was < 25cm and were therefore considered undernourished. Low MUAC was more prevalent in rural dwellers than the urban residents. This could be due to the fact that most rural residents are poor. Poverty has often been associated with increased odds of underweight and individuals who are economically better-off are more likely to gain weight [22]. This is because poverty limits an individual’s access to food to meet daily requirements or ensure dietary diversity, and this could lead to undernutrition [23]. On the other hand, the changing food consumption and physical activity patterns that have led to increasing inactivity especially among urban dwellers and wealthy could be the reason why majority of the study participants had high MUACs [24].

### Determinants of anaemia among respondents

The results of this study showed that dietary diversity score (DDS), maternal age and gestational age are significant determinants of anaemia among pregnant women. This finding corroborates that of [25] conducted among urban dwellers of Northern Ghana that dietary diversity has a positive effect on haemoglobin concentration [25]. The findings of the study however, contradicts the findings of studies carried among pregnant women in Pakistan and rural areas in Northern Ghana [16, 26]. In the present study, a low dietary diversity score increased the odds of anaemia among pregnant women.

Dietary diversity is an indirect way of appraising food availability, accessibility, utilization among individuals and households. Promotion of diverse diets is one of several approaches to improving micronutrient intake for women and children. Consumption of a variety of foods from different food groups provides essential nutrients as well as phytochemicals to the body for normal growth and development as well as prevention of diseases, whilst low dietary diversity leads to malnutrition [27]. In line with the current emphasis on the first 1000 days of an infant’s life, improving the dietary pattern and nutritional status both before and during pregnancy is critical for preventing short- and long-term undesirable effects of malnutrition in pregnancy. In a prospective cohort study, Zerfu [28] recorded reduced risk of maternal anemia, preterm delivery, and low birth weight among rural Ethiopian pregnant women with high dietary diversity score [28].

In this study sample, there was a direct association between gestational age and anaemia. Specifically, respondents in their second trimester of gestation had higher odds of being anaemic than those in their first and third trimesters. It is a well-established fact that due to increased plasma volume, haemoglobin and haematocrit levels decrease during the first trimester and reach the lowest levels at the end of the second trimester and increase again during the third trimester of pregnancy. Similarly anaemia was more prevalent among respondents in their second trimester (52.3%) than those in their first (21%) and third (26.6%) trimesters. The fall in haemoglobin concentration can negatively affect the health the mother if appropriate measures are not taken. The findings of this study is similar to that of Kumar [29].

Results of this study also showed an association between maternal age and anaemia. Particularly respondents within the age bracket of 25 to 34 years had a lower chance of being anaemic than those within the bracket 15 to 24 years. This could be due to the fact that the later age bracket includes teenagers. Apart from the many physiological changes that increase nutrient needs, pregnancy during teen years increase the risk of undesirable effects such as anaemia.

## Limitations of the study

The 24-hour recall was dependent on memory and the ability of the participants to recall accurately. Recall bias could not be ruled out completely because of this. Again, because individuals tend to either overestimate or underestimate their intakes during dietary recalls, the 24-hour dietary recall may not truly represent the usual intake. Although dietary diversity was assessed based on 24-hour recalls which are often associated with recall bias, methods used in assessing dietary diversity are useful for ranking individuals but do not necessarily permit exact assessments of absolute nutrient intake. Additionally, the cross-sectional study design used to collect data also makes it difficult to demonstrate cause-and-effect relationships.

## Conclusion

Generally, 56% of pregnant women had anaemia of any kind. Anaemia prevalence was higher among rural residents than urban residents. The study findings indicate that dietary diversity score, gestational age and maternal age are independent determinants of anaemia among pregnant women. Anaemia prevalence was higher with reduced dietary diversity score, reduced maternal age and increased gestational age. Because dietary diversity is an important tool for improving diet quality and also reduce anaemia risk, it will be necessary for appropriate measures such as nutrition education to be put in place to equip individuals especially pregnant women resident in rural areas make right healthy food choices.
